# Influences of photosensitizer curcumin on microbial survival and physicochemical properties of chicken during storage

**DOI:** 10.1016/j.psj.2022.102417

**Published:** 2022-12-09

**Authors:** Jingwen Gao, Warangkana Srichamnong, Wimonphan Chathiran, Karl R. Matthews

**Affiliations:** ⁎ Department of Food Science, Rutgers University, New Brunswick, NJ 08901, United States; † Institute of Nutrition, Food chemistry Unit, Mahidol University, Tambon Salaya, Thailand

**Keywords:** photosensitizer curcumin, *Listeria monocytogenes*, *Salmonella*, chicken, shelf-life

## Abstract

Curcumin is a natural plant derived antimicrobial, which was shown to inactivate or inhibit the growth of a broad spectrum of microorganisms through photodynamic inactivation. The purpose of the present study is to evaluate the influence of curcumin against commensal spoilage bacteria on chicken, foodborne pathogens, and the chicken skin pH and color. Chicken skin samples were immersed into water, photosensitizer curcumin (**PSC**), or peracetic acid (**PAA**). PSC samples were subsequently subjected to illumination by LEDs (430 nm). The PSC treatments did not inhibit the outgrowth of the four groups of spoilage bacteria evaluated. PSC treatment resulted in 2.9 and 1.5 log CFU/cm^2^ reduction of *L. monocytogenes* and *Salmonella*, respectively. Over a 10-d period, population of *Salmonella* remained significantly lower on PSC treated samples compared to other treatments. PSC treatment resulted in no significant changes in pH or color as compared to water treated samples. This research suggests PSC effectively controlled pathogen outgrowth on chicken without negatively influencing quality; and may be suitable for use in commercial chicken processing.

## INTRODUCTION

Poultry is an important source of protein making it one of the most popular animal proteins consumed globally. Poultry and meat are generally thermally processed before consumption, but they still accounted for the greatest percentage of deaths (29%) associated with foodborne illnesses (Painter et al., 2013). Poultry and meat are perfect matrixes for microbial growth because of near neutral pH, availability of diverse nutrients, and high-water activity. The consumption of poultry meat has increased dramatically over the past 5 decades, exceeding that of beef and pork (Alexandratos and Bruisma, 2012; Mottet and Tempio, 2017). Consumers may undercook poultry (whole carcass or parts) or wash the carcass improperly, leading to cross-contamination and subsequently foodborne illnesses. It was reported that inadequate cooking accounted for over 50% of poultry-associated outbreaks in the United States (Chai et al., 2017). Consequently, poultry safety has become increasingly important.

The shelf-life of a product is important to meeting consumer demands. The outgrowth of bacteria including *Pseudomonas, Enterobacteriaceae,* and lactic acid bacteria can reduce the shelf-life of poultry. Depending on starting population, spoilage bacteria may increase to 8 log CFU/g on chicken when stored at 4°C for several days (Rouger et al., 2017). Indicators of spoilage (off-odor and slime) become evident, too. Research suggests that spoilage bacteria populations on broiler carcasses can be reduced through use of antimicrobial chemicals during commercial processing (Thames et al., 2022).

There are diverse ways to control microbial contamination throughout processing. Chemical interventions are commonly applied to decontaminate poultry carcasses and may include chlorine, peracetic acid, cetylpyridinium chloride, acidified sodium chlorite, organic, and inorganic acids. Chlorine and acids are relatively inexpensive, but high concentrations of chemicals may deteriorate food quality, cause equipment corrosion, pollute the environment and impact consumer health concern. The concept of clean labels and natural antimicrobials has become ever more popular ushering a move away from standard chemical interventions. The present study aims to evaluate the potential of using a novel technology to improve the microbial safety and shelf-life of poultry.

Photodynamic inactivation has been shown to inhibit the growth of a broad spectrum of microorganisms. When a photosensitizer compound is activated by a light source at appropriate wavelength, it is excited from the ground state to the excited state. The molecule in the excited state is unstable, so it releases the excessive energy as heat and light emissions. If the excited molecule goes through the “intersystem crossing,” the excessive energy and/or protons/electrons are released to the surrounding environment, leading to production of reactive oxygen species (**ROS**). ROS are capable of damaging proteins, membrane lipids, and genetic material. The microbial inactivation caused by photodynamic inactivation is non-specific, so it is difficult for microorganisms to develop resistance (Maisch et al., 2005).

Curcumin, a well-known plant derived natural photosensitizer, has attracted a lot of attention for application by the food industry. It is an important phytochemical compound of turmeric (*Curcuma longa*). Numerous studies support the photodynamic inactivation ability of photosensitizer curcumin against a broad spectrum of microorganisms on media, including *Staphylococcus aureus* (Jiang et al., 2014), *Listeria innocua* (de Oliveira et al., 2018), *Vibrio parahaemolyticus* (Wu et al., 2016), *Escherichia coli* O157:H7 (de Oliveira et al., 2018), and *Salmonella* Typhimurium (Penha et al., 2017). Others have evaluated photosensitizer curcumin as an antimicrobial in food models, such as produce (Glueck et al., 2017; de Oliveira et al., 2018), chicken (Tortik et al., 2014; Gao and Matthews, 2020), and oyster (Wu et al., 2015; Wu et al., 2016). Photosensitizer curcumin was found to reduce the level of commensal bacteria on the surface of fruits and oysters prolonging shelf-life (Liu et al., 2016; Lin et al., 2019; Tao et al., 2019; Zhang et al., 2020). Previous research using the photosensitizer curcumin as an antimicrobial treatment for raw poultry did not include influence on shelf-life and food quality (Gao and Matthews, 2020). The purpose of the present study was to determine whether photosensitizer curcumin can extend shelf-life and maintain poultry quality during storage.

## MATERIALS AND METHODS

### Chemicals and Bacterial Strains

Water-dispersible photosensitizer curcumin (PSC; *u*C3 Clear) was kindly provided by Sabinsa Corp. (East Windsor, NJ). PSC solution was prepared by suspending in sterile distilled water (**SDW**). Peracetic acid (**PAA**) solution prepared by diluting a commercial PAA concentrate with SDW, and the PAA concentration was confirmed using test strips (Micro Essential Laboratory Inc., Brooklyn, New York) before each use.

The *L. monocytogenes* and *Salmonella* strains used in this study were obtained from Dr. Joshua Gurtler (Eastern Region Research Center, USDA, Wyndmoor, PA) and Dr. Donald Schaffner (Rutgers University, New Brunswick, NJ), respectively. *Listeria monocytogenes* cocktail contained three strains: L008 (serotype 4b), L2624 (serotype 1/2b), and L2625 (serotype 1/2a). *Salmonella* cocktail included 2 strains associated with poultry: *S.* Hadar and *S.* Heidelberg. Stock cultures were stored in tryptic soy broth (TSB; Difco, Becton Dickinson, Sparks, MD) containing 20% glycerol at −80°C. An isolated colony from tryptic soy agar (TSA; Difco, Becton Dickinson, Sparks, MD) was transferred to 30 mL of brain heart infusion broth (BHI; Difco, Becton Dickinson, Sparks, MD) for *L. monocytogenes* or TSB for *Salmonella*, followed by 20-h incubation at 37°C. To prepare the inoculum cocktail, the culture was washed twice at 4,000 × *g* for 10 min and was sequentially suspended in 0.1% sterile peptone water (SPW; Difco, Becton Dickinson, Sparks, MD) and SDW. Either the three *L. monocytogenes* isolates, or the two *Salmonella* isolates were mixed to achieve a final concentration of approximate 9 log CFU/mL.

### Light Source

The light apparatus was composed of six solderless exotic LED (430 nm; 7.2 W; LED Group Buy, Saint Louis, MO) evenly distributed across the interior top. The power density of the light box unit was measured by taking five readings from the spectroradiometer (Model PS-300, Apogee Instruments, Inc., Logan, UT). The power density of the light apparatus was 107 W/m^2^. Light dose was calculated by multiplying power density by illumination time.

### Preparation of Chicken Skin Samples

Whole chicken carcasses were purchased from a local market and used within 3 h. The chicken breast skin was aseptically removed using a sterile scalpel and cut into 5 × 5 cm^2^ pieces. The skin piece was immobilized on a sterile stainless-steel wire mesh and stored in a sterile petri dish at refrigeration temperature before use. Replicate skin samples were collected from different chicken carcasses.

A 10 μL aliquot of either *L. monocytogenes* or *Salmonella* cocktail was evenly spread on the chicken skin pieces using a sterile plastic spreader. The skin samples were dried for 5 min with the lid of the petri dish ajar and were kept for an additional 25 min with the lid covered to allow attachment.

### Spoilage Bacteria and Chicken Storage

The chicken skin samples were prepared similarly as described previously, except the chicken skin samples were not inoculated with pathogens. Samples were immersed in PAA for 10 s, SDW for 1 min, or PSC for 1 min coupled with activation by the light source (32.1 kJ/m^2^). After treatment, each skin sample was placed in a sterile sample bag containing 25 mL of PBS and stomached for 2 min. The total aerobic plate count (**APC**) was enumerated using plate count agar (Teknova Inc., CA). The level of *Enterobacteriaceae, Pseudomonas* spp., and lactic acid bacteria (**LAB**) were determined by Violet Red Bile Glucose agar (Difco, Becton Dickinson, Sparks, MD), *Pseudomonas* CFC agar (cetrimide-fucidin-cephalosporin; Oxoid, Thermo Scientific), and MRS agar (Difco, Becton Dickinson, Sparks, MD). Media were prepared and incubated as the manufacturer's directions suggested. Samples were collected for microbiological analysis on D 0, D 1, D 3, and D 7. A total of 5 replicated samples were prepared for each treatment/sampling day.

### Listeria Monocytogenes or Salmonella and Chicken Storage

Two skin samples were immersed into 300 mL of a treatment, which had been held at refrigeration temperature to cool the treatment solution to approximate 12°C. PSC solution was protected from natural light by covering the container with aluminum foil. In brief, the PSC-treated (300 ppm, 5 min) samples were exposed to light for 5 min resulting in 32.1 kJ/m^2^ light activation. The water treatment (control) was exposure to SDW, and nontreated samples were not exposed to light (Gao and Matthews, 2020). After treatment, the chicken skin sample was placed in a sterile sample bag containing 25 mL of sterile phosphate buffered saline (PBS; VWR International, LLC). The sample bag was then stomached for 2 min. The levels of *L. monocytogenes* and *Salmonella* were enumerated using modified oxford agar (MOX; Difco, Becton Dickinson, Sparks, MD) and XLT-4 (Becton Dickinson, Sparks, MD), respectively. Each skin sample was placed in a sterile petri dish individually and wrapped with plastic food wrap to mimic home storage. Samples were stored in the refrigerator up to 10 d; they were collected for microbiological analysis (samples collected in triplicate) on D 0, D 1, D 5, and D 10.

### Color and pH of Chicken Skin During Storage

Pieces of chicken skin (n = 5) with approximate 3-mm thickness of flesh were removed using sterile scalpels. Chicken skin samples were treated as described previously (samples were immersed in water or PSC for 1 min, or PAA for 10 s). The color of chicken skin was measured using a colorimeter (Konica Minolta CR410, Osaka, Japan). Five readings from different locations were obtained for each sample. The pH of the chicken skin samples was measured using a pH meter (GeneMate pH meter equipped with pHE-10 electrode; BioExpress), and a total of three readings were obtained for each sample.

### Statistical Analysis

The mean values were compared by ANOVA and Tukey HSD analysis using the SAS software (SAS university edition, SAS Institute Inc., USA). *P* < 0.05 was considered as a significant difference.

## RESULTS AND DISCUSSION

The food industry has embraced sustainability and implementation of practices that serve to achieve sustainability goals. The poultry processing industry utilizes a substantial amount of water and water antimicrobials to wash and rinse carcasses to remove physical debris, reduce microbial load, and improve shelf-life. The use of chemical antimicrobials (e.g., organic and inorganic acids) in wash and rinse solutions for poultry carcasses and parts are not perceived by some consumers as natural or sustainable. Phytochemicals that have antimicrobial properties are more widely accepted by consumers and may be used as part of a multistep process to enhance microbial safety and shelf-life of raw poultry (Quinto et al., 2019). In this study, chicken was sampled on predetermined days for up to 10 d, well beyond the recommended 2 d refrigerated storage of raw chicken (USDA, 2015). The treatment process evaluated in this study for controlling the outgrowth of spoilage bacteria was not effective. PSC treatment of chicken skin resulted in initial reduction of *L. monocytogenes* and *Salmonella* and controlled the outgrowth of *L. monocytogenes* and *Salmonella* during prolonged refrigerated storage. PSC treatment of chicken could be included as one of the final steps in commercial processing of carcasses or parts since a final rinse prior to packaging would not be required.

### Microbial Quality of Chicken Skin During Storage

**Figure 1:**
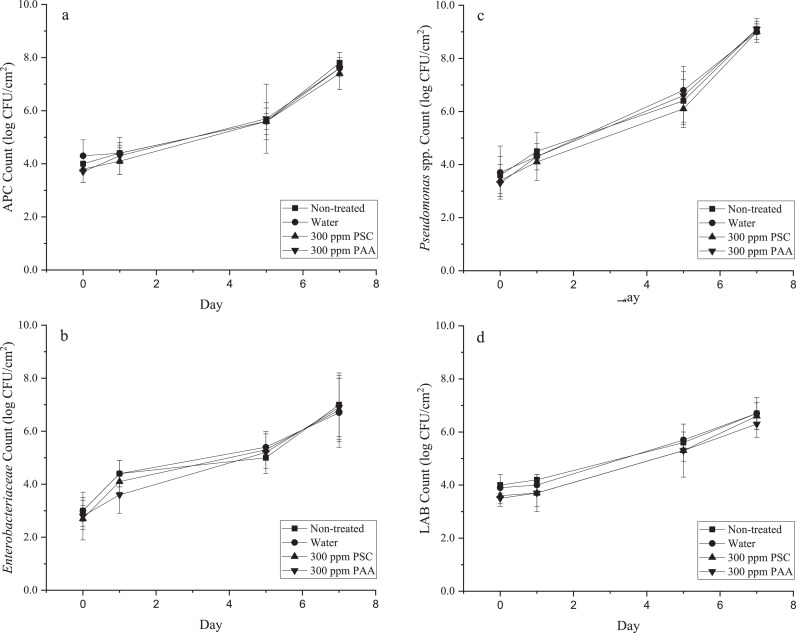
Population of spoilage bacteria under refrigerated storage: (a) APC; (b) *Enterobacteriaceae*; (c) *Pseudomonas* spp.; and (d) lactic acid bacteria. Skin samples were immersed in water or PSC for 1 min, or PAA for 10 seconds on D 0. PSC-treated samples were also exposure to 32.1 kJ/m^2^ after immersion.

Microbial growth is one of the key factors contributing to food spoilage. Spoilage typically occurs when the total aerobic plate count (**APC**) exceeds 7 log CFU/g or the *Pseudomonas* population reaches 8 log CFU/g (Elliott and Michener, 1961; Thames et al., 2022). *Enterobacteriaceae* and lactic acid bacteria are important groups of microorganisms in the natural microbiota of poultry and influence the microbial quality of foods. The treatments evaluated in this study did not inhibit the outgrowth of the four groups of bacteria evaluated (Figure 1). Controlling the outgrowth of food spoilage bacteria may increase product shelf-life. *Pseudomonas* spp. are associated with spoilage of a variety of foods and produce off-odor compounds associated with food spoilage (Russell et al., 1995). In the present study, *Pseudomonas* were not influenced by photodynamic inactivation by PSC (Figure 1c). Another study also showed that pure curcumin had limited antimicrobial activity against *P. aeruginosa* (<0.33 log CFU/mL), suggesting a potential that *Pseudomonas* were more resistant to photodynamic inactivation (Penha et al., 2017). Commensal spoilage bacteria may be present in feather follicles and other structures making it more difficult for the various treatments to contact the bacteria (Lillard, 1988). Commensal bacteria may be associated with biofilms on poultry skin which provides protection from the action of antimicrobials. In this study, a short treatment time was selected since that would be advantageous for high volume commercial poultry processing plants. The sampling schedule for spoilage bacteria was also slightly different from foodborne pathogens since the expectation was, they would multiply more rapidly under refrigeration conditions. Liu et al. (2016) reported that pure curcumin was able to reduce the growth rate of aerobic bacteria on oyster meat, extending the shelf-life of oysters by 4 d when held at 4°C. Variability in efficacy of curcumin in the control of microbial outgrowth on different food commodities is not unexpected and highlights the importance of evaluating specific commodities and treatments.

### Listeria Monocytogenes and Salmonella During Chicken Storage

**Figure 2:**
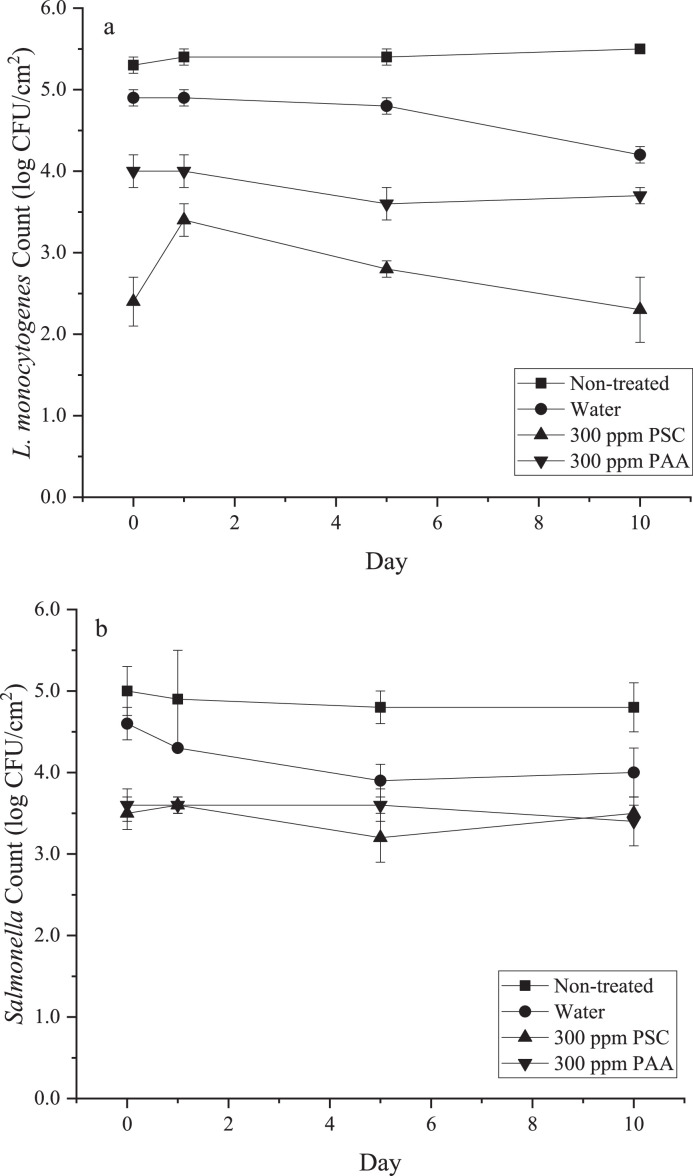
Population of (a) *L. monocytogenes* and (b) *Salmonella* under refrigerated storage. Skin samples were immersed in water or PSC for 5 min, or PAA for 10 s on D 0. PSC-treated samples were also exposure to 32.1 kJ/m^2^ after immersion.

Figure 2 shows the population of *Listeria monocytogenes* and *Salmonella* on chicken skin held under refrigerated storage conditions. PSC treatment resulted in the greatest reduction of each pathogen immediately after treatment. The PSC treatment reduced the population of *L. monocytogenes* by 2.9 log CFU/mL, which was significantly greater than the PAA treatment. Log reduction for PSC treatment against *Salmonella* was not as great as against *L. monocytogenes* but was still significantly greater (*P* < 0.05) than the water treated control. Results suggest that *L. monocytogenes* were more susceptible to photodynamic inactivation than *Salmonella.* This may be related to differences in cell membrane structure and composition of the cell wall. Gram-negative bacteria have an inner and outer cell membrane, while the Gram-positive bacteria have thick peptidoglycan layers, which are more porous than the inner/outer membrane structure. The PSC molecule may better penetrate the Gram-positive bacteria cell membrane, leading to greater disruption of membrane integrity and higher efficacy of photodynamic inactivation (Dai et al., 2009). Results suggest that washing poultry carcasses with photosensitizer curcumin coupled with light activation will reduce the level of pathogen contamination, and thus enhance the safety of poultry.

### Physicochemical Properties of Chicken Skin During Storage

**Figure 3:**
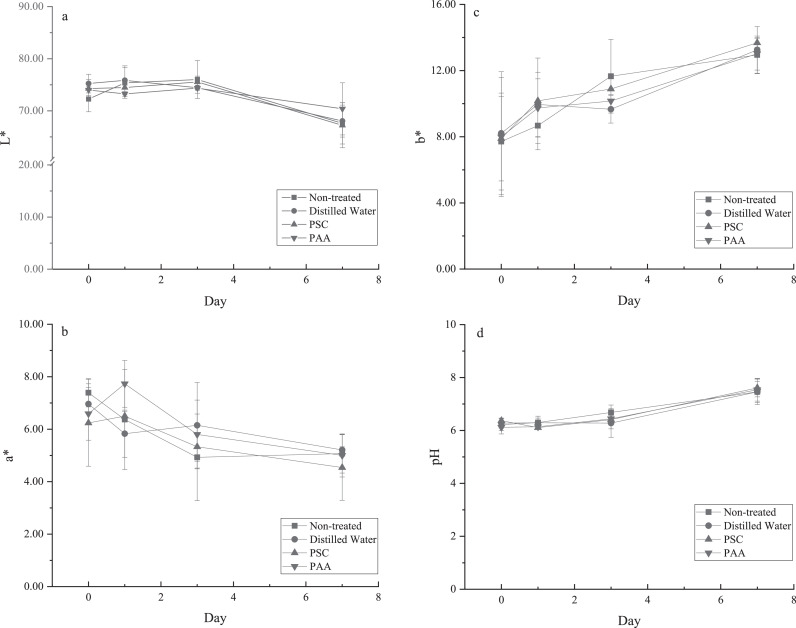
Color (a–c) and pH (d) change in chicken skin under refrigerated storage. Skin samples were immersed in water or PSC for 1 min, or PAA for 10 s on D 0. PSC-treated samples were also exposure to 32.1 kJ/m^2^ after immersion.

Color and pH are important parameters associated with poultry quality and therefore consumer acceptance. Curcumin has a bright yellow color under neutral pH and may be used as a food colorant, but PSC at 300 ppm did not influence the color of the chicken skin when compared with the nontreated and water treated controls (Figure 3a–c). During shelf-life studies chicken skin samples showed a decreasing trend in lightness (L*) and a significant increase in yellowness (b*). Similar results were reported in a previous study for chicken wings stored at 4°C (Kim and Marshall, 2000). Regardless of treatment, the pH of chicken skin significantly increased (*P* < 0.05) by D 7 (Figure 3d). The increase in pH was expected and has been reported previously (Yang and Chen, 1993; Allen et al., 1997; Chun et al., 2010). The increase in pH value may be the result of proteolysis and microbial metabolism leading to production of ammonia, hydrogen sulfide, and other alkaline products (Kakouri and Nychas, 1994; Nychas and Tassou, 1997).

## CONCLUSIONS

The concept of natural has gained popularity with consumers and the food industry fueling an increase in studies evaluating the efficacy of natural plant-based antimicrobials for use in foods. Curcumin is a natural extract of turmeric having suitable properties for use in photodynamic inactivation against a broad spectrum of microorganisms. The present study demonstrated that under conditions evaluated PSC treatment did not negatively influence microbial quality, color, or pH of chicken skin. The treatment use for controlling outgrowth of spoilage bacteria was not effective. PSC treatment provided long-term inhibition in the outgrowth of *L. monocytogenes* and *Salmonella* on chicken skin held under refrigeration storage conditions. The efficacy of PSC in controlling *L. monocytogenes* and *Salmonella* was equivalent or better than treatment using 300 ppm of peracetic acid. The results of the present study are encouraging and suggest the photosensitizer curcumin has potential application in the treatment of poultry carcasses and parts to improve microbial safety.
